# Protective Effects of Triphala on Dermal Fibroblasts and Human Keratinocytes

**DOI:** 10.1371/journal.pone.0145921

**Published:** 2016-01-05

**Authors:** Sandeep R. Varma, Thiyagarajan O. Sivaprakasam, Abheepsa Mishra, L. M. Sharath Kumar, N. S. Prakash, Sunil Prabhu, Shyam Ramakrishnan

**Affiliations:** Research and Development, The Himalaya Drug Company, Bangalore 562 162, India; University of Alabama at Birmingham, UNITED STATES

## Abstract

Human skin is body’s vital organ constantly exposed to abiotic oxidative stress. This can have deleterious effects on skin such as darkening, skin damage, and aging. Plant-derived products having skin-protective effects are well-known traditionally. Triphala, a formulation of three fruit products, is one of the most important rasayana drugs used in Ayurveda. Several skin care products based on Triphala are available that claim its protective effects on facial skin. However, the skin protective effects of Triphala extract (TE) and its mechanistic action on skin cells have not been elucidated *in vitro*. Gallic acid, ellagic acid, and chebulinic acid were deduced by LC-MS as the major constituents of TE. The identified key compounds were docked with skin-related proteins to predict their binding affinity. The IC_50_ values for TE on human dermal fibroblasts (HDF) and human keratinocytes (HaCaT) were 204.90 ± 7.6 and 239.13 ± 4.3 μg/mL respectively. The antioxidant capacity of TE was 481.33 ± 1.5 mM Trolox equivalents in HaCaT cells. Triphala extract inhibited hydrogen peroxide (H_2_O_2_) induced RBC haemolysis (IC_50_ 64.95 μg/mL), nitric oxide production by 48.62 ± 2.2%, and showed high reducing power activity. TE also rescued HDF from H_2_O_2_-induced damage; inhibited H_2_O_2_ induced cellular senescence and protected HDF from DNA damage. TE increased collagen-I, involucrin and filaggrin synthesis by 70.72 ± 2.3%, 67.61 ± 2.1% and 51.91 ± 3.5% in HDF or HaCaT cells respectively. TE also exhibited anti-tyrosinase and melanin inhibition properties in a dose-dependent manner. TE increased the mRNA expression of collagen-I, elastin, superoxide dismutase (SOD-2), aquaporin-3 (AQP-3), filaggrin, involucrin, transglutaminase in HDF or HaCaT cells, and decreased the mRNA levels of tyrosinase in B16F10 cells. Thus, Triphala exhibits protective benefits on skin cells *in vitro* and can be used as a potential ingredient in skin care formulations.

## Introduction

The skin of the human beings acts as a bridge between internal and external environment being well-equipped with an intricate network of antioxidant substances (redox-active) and enzymes, continually exposed to the oxidative environment [1, 2]. The skin is composed of three layers: epidermal, dermal, and subcutaneous, out of which epidermis is adversely affected by abiotic factors [3]. The environmental stress along with normal aging process can deplete the epidermis of protective antioxidants leading to skin damage, darkening, and premature aging. Several extrinsic factors enhance the skin aging process like UV radiation, environmental pollution, poor nutrition, and smoking that contribute to premature aging [4]. The cellular redox process constantly produces free radicals like reactive oxygen species (ROS) as well as reactive nitrogen species (RNS) by normally using body’s oxygen [4]. Also, exposure to UV radiation leads to increase in ROS/RNS production, fatty acids peroxidation with formation of hydroperoxides [3]. These free radicals possess unusual attraction for biological macromolecules (DNA, RNA, and proteins) leading to oxidative damage [5]. The specific signs of skin aging occur due to several biological mechanisms, which include loss of skin structural proteins, loss of hydration, and increased sensitivity to UV-induced pigmentation. The biological phenomena of skin aging are associated with specific aging-related genes referred as functional youth gene clusters [6]. All the above phenomena has led to adaption of skin by developing inbuilt mechanism to protect, restore or maintain proper balance with respect to harsh environmental conditions [1]. Phytochemicals from natural sources are extensively utilized in conventional Indian medicinal practices for improving the skin condition and restoring its vitality. For example; melanin inhibitors (hydroquinone, arbutin, and kojic acid) derived from plants is used in cosmetics for protection against skin darkening [7–9]. Phytoactives, dietary components, and food ingredients have protective effects on skin playing active role in cosmeceuticals. The formulation consisting of one or more phytoactives have been utilized in Indian ayurvedic system for more than 5,000 years to address various medical or skin conditions. Triphala is one such well-favored herbal formulation used worldwide for various health benefits [10].

Triphala, formulated as powder of three myrobylan fruits, *Emblica officinalis* Gaertn, *Terminalia chebula* Retz, and *Terminalia belerica* Roxb in equal proportions exhibits plethora of health benefits [11–12]. Several research papers validate the ethnomedicinal claims that Triphala displays free radical-scavenging, antioxidant, anti-inflammatory, antipyretic, analgesic, antibacterial, antimutagenic, wound-healing, anticarcinogenic, antistress, adaptogenic, hypoglycaemic, anticancer, radioprotective, chemoprotective, and chemopreventive properties [13–14]. Most of the health properties of Triphala have been attributed to the presence of polyphenolic secondary metabolites such as gallic acid, ellagic acid, and chebulinic acid, the major constituents of Triphala [12–15]. Triphala powder is traditionally applied on the face to reduce the physiological signs of aging. However, the detailed biological mechanism behind its skin protection action is not well investigated at cellular and molecular level.

The objective of the study was to investigate the skin protective benefits of Triphala on human dermal fibroblasts (HDF) and human keratinocytes (HaCaT) cells.

## Materials and Methods

### Maintenance of cell lines

Human Dermal Fibroblast (HDF), Human Keratinocytes (HaCaT), Mouse melanoma (B16F10), Human melanoma (A-375), and Mouse monocyte macrophage (RAW264.7) cells were procured from American Type Culture Collection (ATCC). HDF cells were cultured in minimum essential medium (MEM), HaCaT cells were cultured in Ham’s F-12 medium, and RAW264.7/A-375 cells were cultured in Dulbecco’s modified Eagle’s medium (DMEM) high glucose medium, with 10% fetal bovine serum, penicillin (100 U/ml), and streptomycin (100 μg/ml) at 37°C in a humidified CO_2_ (5%) chamber_._

### Chemicals and reagents

Fetal Bovine Serum (FBS), 3-(4,5-dimethylthiazol-2-yl)-2,5-diphenyl tetrazolium bromide (MTT), dimethyl sulphoxide (DMSO), trichloroacetic acid (TCA), N-(1-naphthyl) ethylenediamine dihydrochloride, sulphanilamide, ascorbic acid, tannic acid, custom oligonucleotides were obtained from Sigma-Aldrich, MO, USA. Penicillin, streptomycin, potassium ferricyanide, H_2_O_2_, ferric chloride, ferrous sulphate (FeSO_4_), and sodium nitrite were obtained from Himedia (Mumbai, India). Cellular Senescence Assay kits were purchased from Millipore Corp. (USA). Involucrin and filaggrin ELISA kits were purchased from Cloud-Clone Corp. (USA), Collagen-Type I kits were purchased from Blue Gene Biotech, China. All molecular biology reagents for PCR were obtained from Bio Rad, USA.

### Composition and preparation of Triphala extract

Triphala contains equal proportions of three fruits- Indian gooseberry (*Phyllanthus emblica* L.), Haritaki (*Terminalia chebula* Retz.), and Vibhitaki (*Terminalia bellerica* Roxb.). The drug regulatory authority department of AYUSH have recommended Triphala as natural medicine approved by Ministry of Health and Family Welfare, Government of India. The herbs used in the formulation were procured following good procuring practices and well-maintained in proper conditions. The plants were taxonomically classified by Dr. Kannan, a pharmacognosist and sample of plant constituents has been deposited in the herbarium of R&D, The Himalaya Drug Company, Bangalore, India. Triphala granules (100 g) (Batch No. 1308032 FD) were crushed in liquid nitrogen using mortar and pestle to obtain fine powder. It was sequestered with 1000 ml of water at room temperature for 30 min. The mixture was then extracted at 37°C for 24 h in a shaking water bath (300 rpm). The slurries were cooled down to room temperature, centrifuged at 1000 rpm for 15 min, and the supernatant was collected. The extract was lyophilized and a yield of 2.9% of the DW of Triphala was obtained. This Triphala extract (TE) was used for all the experimental studies.

### Phytochemical analysis

The total phenol content, tannin, and flavonoid content in TE was estimated using spectrophotometric or colorimetric methods as described earlier [16–18]. The Folin-Ciocalteau and Folin-Denis method was followed for determination of phenol and tannin content. The total flavonoid content was measured colorimetrically in Triphala extract (TE) using quercetin as standard. Gallic acid and Quercetin were used as reference standards in the experiments.

### Liquid chromatography-mass spectrophotometer (LC-MS) analysis

The extracts of Triphala (TE) and its individual constituents were subjected to LC-MS analysis. The sample was prepared in water and filtered through 0.2 μm filter prior to injection into the mass spectrometer. Sample was injected into the ES ionization source using a syringe pump at a flow rate of 30μl/min.

LC-MS conditions: The API 2000 (Applied biosystem/MDS SCIEX, Canada) mass spectrometer coupled with an (Electron spray ionization) source and a HPLC (Shimadzu LC-20AD) was used for molecular mass determination and analysis of Triphala and its individual constituents extracts. The instrument was operating in the negative mode with an ion spray voltage -4500 V and declustering potential -20 V. The analyst 1.5 version software was used for controlling batch acquisition and data analysis [19].

### Molecular docking studies

Molecular docking was performed with energy minimized structures of protein and ligands. The energy-minimized structure of gallic acid, ellagic acid, and chebulinic acid were docked with AQP-3, SOD-2, Collagen, Transglutaminase, Involucrin, Filaggrin, and Tyrosinase using AutoDock Vina 1.1.2 [20]. The protein and ligand files were converted into a PDBQT file format, a modified protein data bank format having atomic charges and definitions for ligands including topological information. After receptor–ligand preparation and specifying the binding site, program for docking was run from the command prompt [21]. The binding energy was calculated for the docked complex (Receptor-ligand) and represented as affinity (kcal/mol).

### Cell cytotoxicity assay

HDF and HaCaT cells were cultured in 96-well plates (1 × 10^4^ cells/ml) and treated with various concentrations (15.82–1000 μg/ml) of TE respectively. The percentage cytotoxicity was estimated by MTT assay at 540 nm after 24 h incubation, [22] using the Synergy HT multi-detection microplate reader (Bio-Tek, Winooski, VT). From the values obtained, the percentage cytotoxicity (IC_50_ value) was calculated. For further experiments, non-toxic concentrations were used in the study.

### RBC hemolysis assay

The RBC hemolysis was performed with the modified protocol originally described by Reddy et al [23]. The volume of TE samples and control with erythrocyte suspensions were adjusted to 1 ml by adding 0.9% saline. H_2_O_2_ (100 μM) was used to induce oxidative stress and the samples were incubated at 37°C for 3 h. After incubation, the mixture was centrifuged at 1200 rpm for 5 min followed by supernatant collection. The absorbance was measured at 540 nm using Synergy HT multi-detection microplate reader (Bio-Tek, Winooski, VT). RBC hemolysis by H_2_O_2_ was taken as 100% cell lysis, and hemolysis of the treated and untreated erythrocytes was calculated as percentage of this value. Ascorbic acid (100 μM) served as positive control

### Reducing power assay

Various concentrations of TE (0–400 μg/ml) in corresponding solvents were mixed with 500 μl phosphate buffer (pH 6.6) and 500 μl of 1% potassium ferricyanide. The mixture was incubated at 50°C in water bath for 20 min. After cooling, 400 μl of 10% TCA was added and centrifuged at 10,000 rpm for 20 min. The supernatant (500 μl) was mixed with 500 μl of distilled water and 100 μl of a freshly prepared 0.1% ferric chloride solution. The absorbance was read at 700 nm [24]. In a similar way, control was also prepared excluding the test samples. Ascorbic acid at various concentrations (0–400 μg/ml) was used as standard.

### Antioxidant assay

HaCaT cells (1 × 10^4^ cells/ml) were seeded in 40-mm petri plates and incubated for 24 h at 37°C. The cells were treated with two concentrations of TE (50 and 100 μg/ml) and incubated for 24 h at 37°C with 5% CO_**2**_. The cell lysates were collected and the antioxidant capacity was analyzed using the total antioxidant power kit (Oxford Biomedical Research, USA). The analysis was repeated thrice (n = 3) by comparing and interpolating the values with that of the standard 6-hydroxy-2, 5, 7, 8-tetramethylchroman-2-carboxylic acid (Trolox) from the calibration curve.

### NO-scavenging assay

RAW264.7 cells were seeded into 40-mm Petri plates (1 × 10^6^ cells) and incubated for 24 h at 37°C. The cells were treated with TE (50 and 100 μg/ml) and lipopolysaccharide (LPS; 1 μg/ml) and further incubated for 24 h at 37°C. NO production was measured in the culture medium and detected by the Griess reaction using sodium nitrite as the standard. Griess reagent (0.1% N-(1- naphtyl) ethylenediamide dihydrochloride, 1% sulphanilamide in 5% phosphoric acid) and an equal volume of cell supernatants were mixed and the absorbance was measured at 540 nm. The nitrite concentration was calculated and expressed as μmol/ml. Dexamethasone (100 μM) was used as standard in this experiment.

### Radical scavenging assay

HDF cells were used for the radical scavenging assay and the protocol described by Murrell *et al* [25] was slightly modified. Fibroblasts were seeded at 1 × 10^**4**^ cells/well in 96-well plates and grown in MEM and incubated for 24 h at 37°C. Three different experimental sets were performed: a) Addition of TE along with H_2_O_2_ to cells (co-treatment); b) Pre-treatment of cells with TE for 1 h prior exposure to the H_2_O_2_, and c) Post-treatment with TE after H_2_O_2_ exposure for 1 h [26]. Ascorbic acid (100 μg/ml), a H_2_O_2_ scavenger was used as a reference standard. Cell control and cells treated with H_2_O_2_ alone were also maintained. The plates were incubated at 37°C for 24 h and MTT assay was used to assess the protection offered by TE.

### DNA protection assay

For investigating the beneficial effects of TE on hydroxyl (OH) radical induced DNA damage, total DNA was isolated from HDF using the DNA isolation kit (Krishgen Biosystems, India). The reaction was performed in a tube containing 3 μl of DNA in 3 μl of PBS (pH 7.4), which was pre-treated with 2 μl of TE at two concentrations (50 and 100 μg/ml) and incubated at 37°C for 1 h. Later, 2.5 μl of 2 mM FeSO_4_ and 7.5 μl of 1 mM H_2_O_2_ were added; the mixture was incubated at 37°C for 30 min [27]. After incubation, the mixture was loaded on to 1% agarose gel and stained with 1 mg/ml ethidium bromide before visualization. DNA bands were observed under the UV light. Densitometric analysis was carried out using Image J software (Rasband, USA) and a graph was plotted.

### Cellular senescence assay

HDF cells (1 × 10^4^ cells/ml) were seeded into 40-mm Petri plates and incubated for 24 h at 37°C. The cells were co-treated with TE (50 and 100 μg/ml) and H_2_O_2_ (10 μM) for 1 h at 37°C. The media was aspirated and the cells were rinsed twice with 2 ml of phosphate buffered saline (PBS). The cells were stained with SA-β-gal as described by the cellular senescence assay kit. The cells were incubated overnight at 37°C without CO_2,_ away from light. The stained cells were rinsed twice in 2 ml of PBS and were scored under light microscope (10×).

### Tyrosinase inhibition assay

The mouse melanoma cells (B16F10) were cultured in the 40 mm petri plates (1×10^5^ cells/well) for 24 h. The cells were treated with or without several concentrations of TE and further stimulated for 24 h in the presence or absence of 100 nM α-MSH. The lysis buffer (100 μL of 50 mM sodium phosphate buffer (pH 6.8) with 1% Triton X-100 and 0.1 mM PMSF) was used to lyse cells and it was further centrifuged at 20,000 × g for 30 min at 4°C. The supernatant collected was added to the plate and the ELISA plate reader was used to monitor absorbance at 492 nm [28–30]. The percentage of enzyme inhibition was computed according to the given formula: Percent inhibition (%) = [C − T/C] × 100, where C and T are the absorbance for the control and test samples, respectively [31]. For standard tyrosinase inhibitor, Kojic acid (100 μM) was used in the experiments.

### Melanin spitting/inhibition assay

The mouse melanoma (B16F10) and Human melanoma (A-375) cells were seeded in the 40 mm petriplates (1×10^5^ cells/well) using the DMEM medium supplemented with 10% FBS for 24 h. The cells were treated with various concentrations of TE and 100 μM concentration of forskolin for the stimulation of melanin for 72 h and detached by 0.05% trypsin-EDTA. The cell pellets were mixed in 120 μL of 1 N NaOH for 1 h at 65°C and melanin content was read at 405 nm through an ELISA reader [30–31]. For melanin content analysis, kojic acid was used as a reference standard.

### Collagen-I, involucrin, and filaggrin ELISA

HDF or HaCaT cells were used for collagen-I, involucrin, and filaggrin estimation, respectively. The cells (1 × 10^4^ cells/ml) were seeded into 40-mm Petri plates and incubated for 24 h at 37°C. The cells were treated with TE (50 and 100 μg/ml) and incubated for 96 h at 37°C_._ Ascorbic acid and Kinetin were used as positive controls for collagen-I, involucrin, and filaggrin ELISA respectively. After incubation period, the supernatant was collected and collagen-1, involucrin, and filaggrin content was estimated using commercially available ELISA kits. For visualization of collagen-I, TE treated HDF cells were stained with Sirus red [32] and the images were captured using phase contrast microscope.

### Real time PCR

To further validate the role of TE on skin differentiation and/or youthful markers, HDF and HaCaT cells (1 × 10^6^ cells/ml) were cultured in 60-mm dishes. The cells were treated with two concentrations of TE (50 and 100 μg/ml) and incubated for 24 h at 37°C. Cell control was also maintained. Total RNA extraction was performed on both the cells using RNA isolation kits (Krishgen Biosystems). The total RNA was treated with DNase to avoid contamination from the genomic DNA. For real-time quantitative PCR (qPCR), 1 μg of total RNA was reverse transcribed into first-strand cDNA using SuperScript III (invitrogen) and an oligo-dT primer. The PCR amplification occurred in a reaction volume of 20 μl containing 2 μL cDNA, 10 μl SYBR Green supermix (Bio Rad, USA), and CFX96 real-time system (Bio Rad, USA). The primers used for collagen-I, elastin, AQP-3, SOD-2, involucrin, filaggrin, transglutaminase (TGM1), and tyrosinase are listed in Table 1. The mRNA expression levels were normalized to the level of glyceraldehyde 3-phosphate dehydrogenase (GAPDH) expression. The Ct values of TE treated cells were calculated and the data was expressed in terms of fold change over cell control. Also, the amplified product was run in 1.5% agarose gel electrophoresis and gel picture captured under UV light.

**Table 1:** List of primers used in RT-PCR studies.

Gene	Primer sequence	Annealing Temperature
**Col-I**	**For 5'-TGACGAGACCAAGAACTG-3'**	**60°C**
	**Rev 5’-TACCAGGGTTTGAGCTCAGC-3’**	
**Elastin**	**For 5’- GCCCCTGGATAAAAGACTCC-3’ Rev 5’- GTCCTCCTGCTCCTGCTGT-3’**	**60°C**
**AQP-3**	**For 5’- GGTTGATGGTGAGGAAACCA-3’ Rev 5’- GGGACCCTCATCCTGGTG- 3’**	**60°C**
**SOD-2**	**For 5’- CGACCTGCCCTACGACTACG- 3’ Rev 5’- TGACCACCACCATTGAACTT-3’**	**60°C**
**TGM1**	**For 5’- CCCCCGCAATGAGATCTACA- 3’ Rev 5’- ATCCTCATGGTCCACGTACACA-3’**	**60°C**
**Filaggrin**	**For 5’- TTTCGTGTTTGTCTGCTTGC- 3’ Rev 5’- CTGGACACTCAGGTTCCCAT-3’**	**60°C**
**Tyrosinase**	**For 5’- GGCCAGCTTTCAGGCAGAGGT- 3’ Rev 5’- TGGTGCTTCATGGGCAAAATC-3’**	**60°C**
**Involucrin**	**For 5’- ACTGAGGGCAGGGGAGAG- 3’ Rev 5’- TCTGCCTCAGCCTTACTGTG-3’**	**60°C**
**GAPDH (Human)**	**For- 5’ AAGGTGAAGGTCGGAGTCAA 3’ Rev- 5’ AATGAAGGGGTCATTGATGG 3’**	**60°C**
**GAPDH (Mouse)**	**For- 5’ ACCACAGTCCATGCCATCAC 3’ Rev- 5’ TCCACCACCCTGTTGCTGTA 3’**	**60°C**

### Statistical analysis

All the experiments were performed in triplicates and every data point indicates the standard deviation of three samples. Graphpad Software QuickCalcs Student's t-test was used for data evaluation and also for computing the significance level of P < 0.05 and P < 0.01.

## Results and Discussion

Over the years, several skin protecting agents of natural and synthetic origin have been introduced [33]. Natural herbal compounds like phenolic acids, flavonoids, and high molecular weight polyphenols have attracted huge attention as beneficial protective agents [34]. Triphala, the magical wonder from the Ayurveda is conventionally used for treating various skin conditions like skin aging, since antiquity [35]. A potential skin protecting compound can control the ROS and oxidative stress, promote exfoliation, skin turnover, and help in maintaining consistent synthesis and replacement of molecules associated with extracellular matrix [36]. It is well–known fact that free radicals play a crucial role in the progression of some diseases including aging. It is also clear that supplementing the human body with antioxidants helps in oxidative stress reduction and slows down problems related to diseased conditions [37]. Several plant phytoactives demonstrate free radical-scavenging or antioxidant activity [38].

The present study explored the natural antioxidant and skin protective properties of Triphala (TE) in quenching the reactive oxygen and nitrogen radicals, delaying cellular senescence and regulating marker genes involved in various biological process of aging. In addition, the key constituents of Triphala responsible for its skin protective role were also identified by mass spectrometry and their functions were predicted by molecular docking prior to *in vitro* studies.

**Fig 1:**
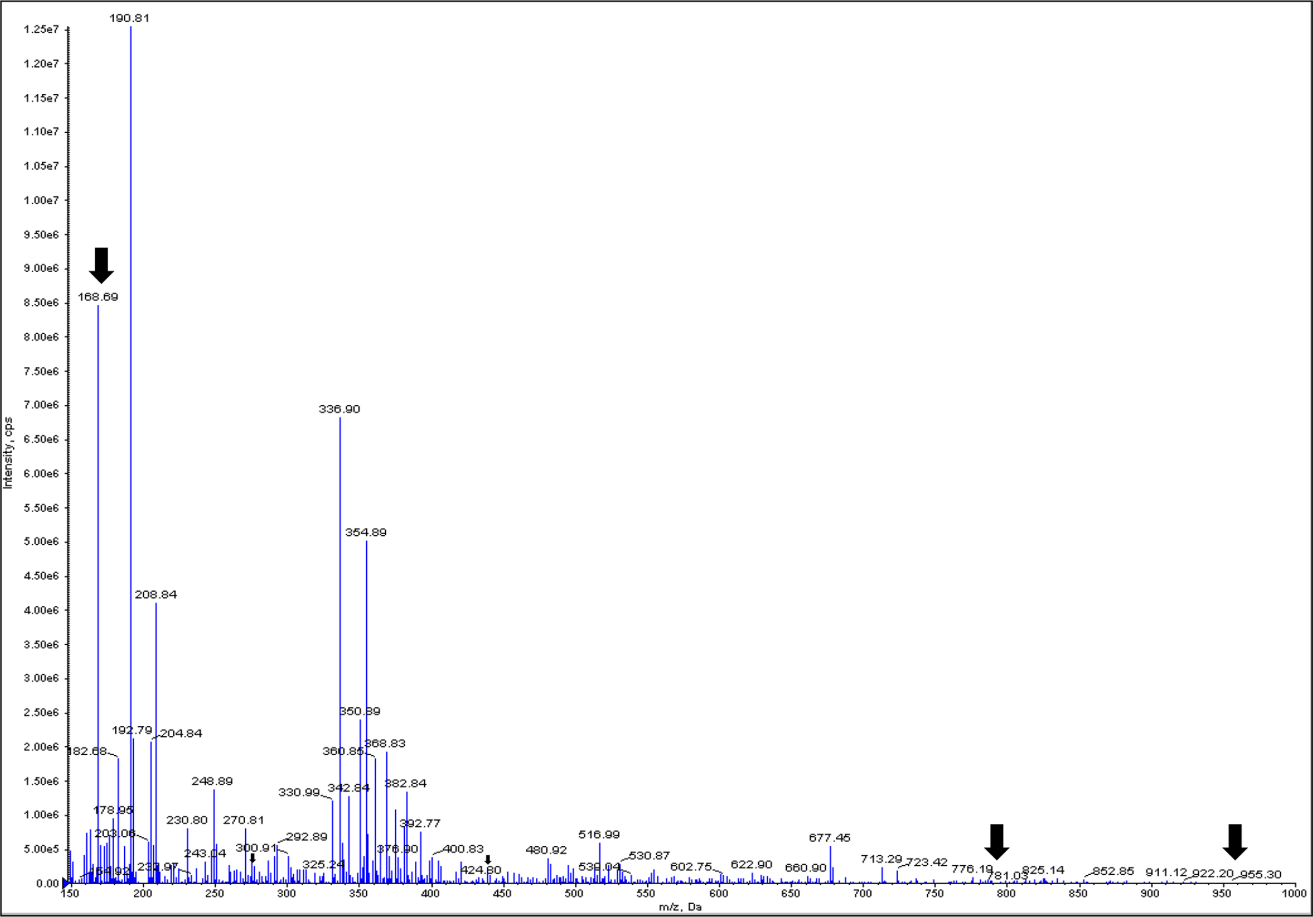
LC-MS/MS characteristics of Triphala and its constituents. The Triphala extracts (TE) were subjected to LC-MS analysis. Sample was injected into the ES ionization source using a syringe pump at a flow rate of 30μl/min. LC-MS/MS conditions are described in the text. The mass spectrum was acquired in the negative mode with a voltage of -4500 V and declustering potential -20 V. All the batch acquisition and data processing were controlled by Analyst 1.5 version software.

Triphala (TE) and its individual constituents were subjected to LC-MS for identifying the key compounds contributing to its biological activity. The presence of gallic acid, and ellagic acid were detected in both TE (Fig 1) and its individual constituents Table 2 (S1–S3 Figs).

**Table 2:** LC-MS/MS characteristics of Triphala and its constituents.

Compound Name	Molecular formula	Molecular weight	Obtained molecular ion
		(g/mol)	(m/z)
***Terminalia chebula***			
**Gallic acid**	**C**_**7**_**H**_**6**_**O**_**5**_	**170.12**	**168.92**
**Ellagic acid**	**C**_**14**_**H**_**6**_**O**_**8**_	**302.19**	**300.96**
**Chebullagic acid**	**C**_**41**_**H**_**30**_**O**_**27**_	**954.66**	**953.1**
**Chebulinic acid**	**C**_**41**_**H**_**32**_**O**_**27**_	**956.67**	**954.97**
***Emblica officinalis***			
**Gallic acid**	**C**_**7**_**H**_**6**_**O**_**5**_	**170.12**	**168.83**
**Emblicanin A & B**	**C**_**34**_**H**_**22**_**O**_**22**_	**782.52**	**781.2**
**Geraniin**	**C**_**41**_**H**_**28**_**O**_**27**_	**952.64**	**952.67**
***Terminalia bellerica***			
**Gallic acid**	**C**_**7**_**H**_**6**_**O**_**5**_	**170.12**	**168.78**
**Friedelin**	**C**_**30**_**H**_**50**_**O**	**426.71**	**424.9**
**Ellagic acid**	**C**_**14**_**H**_**6**_**O**_**8**_	**302.19**	**300.81**
**Triphala extract (TE)**			
**Gallic acid**	**C**_**7**_**H**_**6**_**O**_**5**_	**170.12**	**168.69**
**Ellagic acid**	**C**_**14**_**H**_**6**_**O**_**8**_	**302.19**	**300.91**
**Chebulinic acid**	**C**_**41**_**H**_**32**_**O**_**27**_	**956.67**	**955.3**
**Emblicanin A & B**	**C**_**34**_**H**_**22**_**O**_**22**_	**782.52**	**781.03**
**Friedelin**	**C**_**30**_**H**_**50**_**O**	**426.71**	**424.8**

Recently, the metabolite profiling for polyphenols in *T*.*chebula* showed the presence of several compounds including chebulinic acid [39]. Also, the HPLC profile of Triphala showed gallic acid, ellagic acid, and chebulinic acid to be the predominant compounds [40]. The compounds identified above were docked with various skin-related proteins and represented as binding affinities in Kcal/mol (S1 Table). In addition, the docking studies of major compounds of TE with involucrin, filaggrin, and collagen-I are shown in (S4 Fig). The binding affinity closer to 10 and above indicated efficient binding. It is predicted that binding of ligand with the protein could either inhibit or enhance its activity.

A preliminary phytochemical analysis revealed TE as a rich source of polyphenols (34.6 ± 3%) and tannins (32.5 ± 2%). It also indicated the negligible flavonoid content in TE. Previously, also the phytochemical analysis of the Triphala revealed polyphenols and tannins to be the major constituents [18]. In order to establish the protective role of TE on skin, cellular cytotoxicity was performed using MTT assay, a technique to monitor the cellular health of the cells. The IC_50_ values for TE on HDF and HaCaT were 204.90 ± 7.6 and 239.13 ± 4.3 μg/mL respectively. Only higher concentrations of TE were cytotoxic to both HDF and HaCaT cells (S5 Fig). Thus the non-toxic concentrations were further used for subsequent *in vitro* experiments.

**Fig 2:**
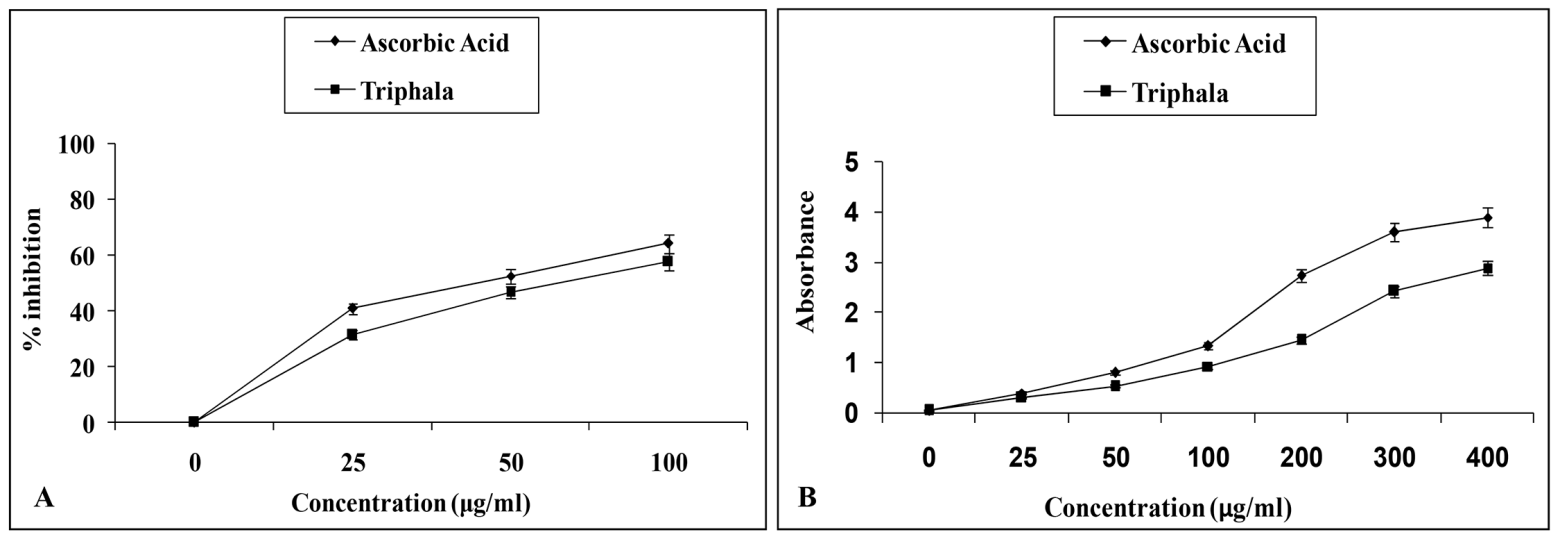
(A) Effect of TE on H_2_O_2_-induced RBC hemolysis. RBC cells were treated with different concentrations of TE and ascorbic acid (AA) (25–100 μg/mL) and the cells were induced with 100 μM H_2_O_2_ for cell lysis. Data was expressed as percentage of control. Results are shown as mean ± SEM of three experiments. (B) Reducing power activity of TE at different concentrations (0–400 μg/ml). Absorbance was read at 700 nm and the data are represented as ascorbic acid equivalents. Results expressed as mean ± SEM (n = 3).

A hemocompatibility study was conducted to validate the cytotoxic effects of TE on normal RBC cells. TE dose dependently inhibited RBC haemolysis with 57.87 ± 0.8% inhibition at 100 μg/ml with IC_50_ values of 64.95 μg/ml (Fig 2A), whereas ascorbic acid (100 μg/ml) inhibited RBC haemolysis by 63.60 ± 0.9%. H_2_O_2_ (control) showed complete (100%) haemolysis. Erythrocytes become frangible in adverse conditions of H_2_O_2_ exposure; it triggers a multitude of changes at cellular level like alteration in membrane deformation, hemolysis, rearrangement of phospholipids, cell surface morphology, and erythrocytes alteration [41–43]. Thus, RBC hemolysis assay demonstrated the effectiveness of TE where it displayed no significant lysis of normal RBC cells compared to the control. TE established to be an effective antioxidant by protecting RBCs from membrane damage due to increasing H_2_O_2_-induced oxidative stress.

The effective antioxidant capacity of TE was evaluated using reducing power assay. In reducing power assay, transfer of electrons occurs wherein a ferric salt is utilized as an oxidant [44].The reducing ability of TE highlighted its antioxidant potential (Fig 2B). Throughout the concentration range (0–400 μg/ml), TE and Ascorbic acid showed nearly the same trend in their reductive capability. The reducing power of TE at 400 and 25 μg/ml was equivalent to that of ascorbic acid at 216.86 ± 5.1 μg/mL and 18.01 ± 0.5 μg/mL respectively. Antioxidant substances are present as reductant in the antioxidant samples leading to reduction of the Fe3+/ferricyanide complex to the ferrous form. The formation of Fe2+ can be measured by observing the development of Perl’s Prussian blue color at 700 nm [44]. The reducing capacity of TE demonstrates its potential as an antioxidant.

The problem in measurement of individual antioxidant substances and possible synergy among antioxidants, has led to the development of methods to assess the total antioxidant capacity of biological specimens [45]. The total cellular antioxidant capacity of TE was evaluated using HaCaT cells. The antioxidant capacity of TE was found to be 481.33 ± 1.5 mM Trolox equivalents in HaCaT cells. It was earlier reported that human epidermis has more antioxidant capacity compared to dermis [46]. The total antioxidant activity of the TE was evaluated by comparing with the standard solution of Trolox.

**Fig 3:**
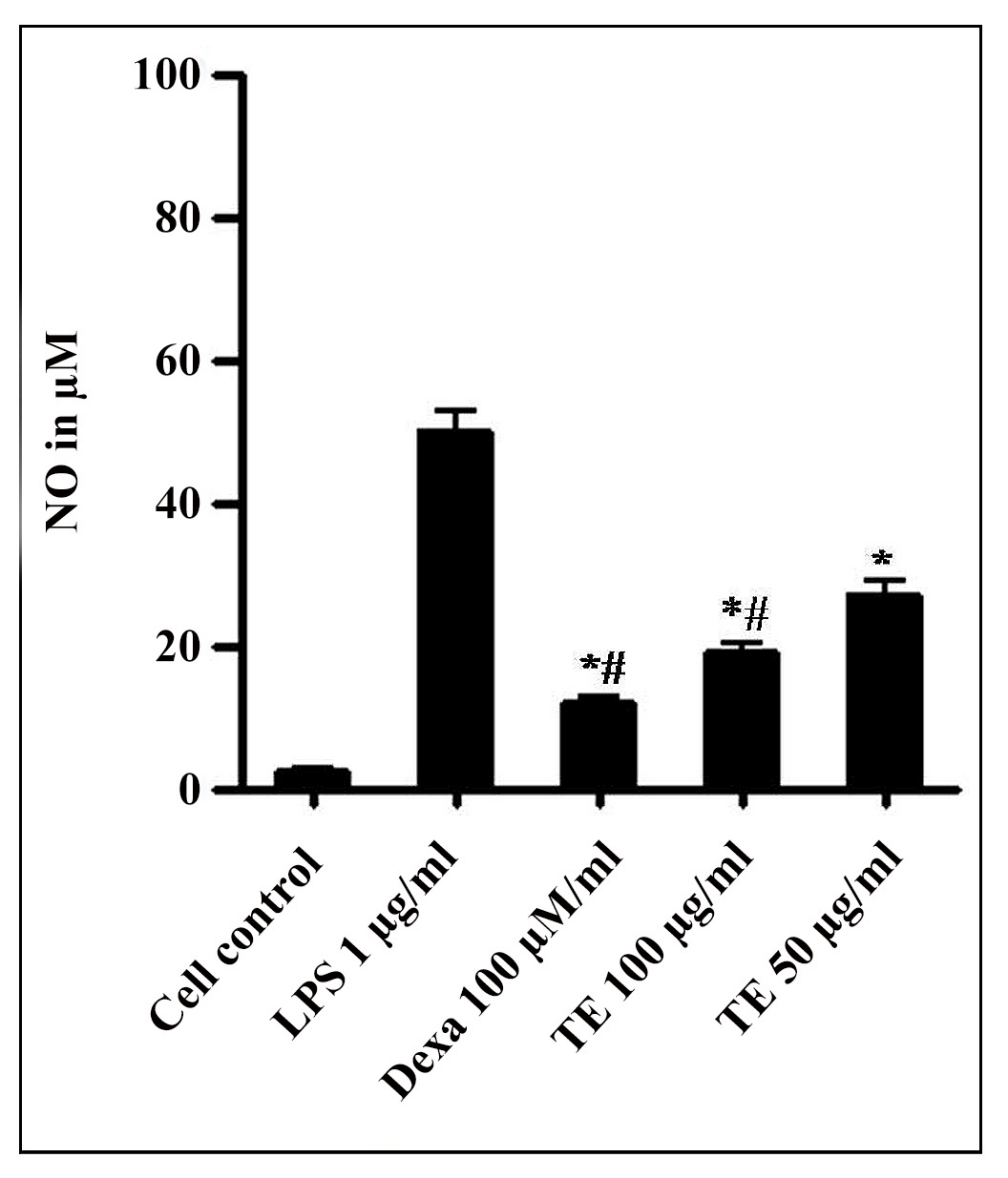
Effect of TE on NO production in LPS-stimulated RAW264.7 cells. Cells were treated with TE (50 and 100 μg/mL) and dexamethasone (Dexa) (100 μM), with/without LPS (1μg/mL) and incubated for 24 h at 37°C. After incubation, the cell supernatant was used to determine NO level by Griess reagent method. The values are expressed as mean ± SEM of three experiments (*p* ≤ 0.01, p ≤ 0.05).

Nitric oxide (NO), a bioactive molecule plays vital roles in physiological and pathological processes [47]. To evaluate the effect of TE in curbing NO production, nitric oxide assay was performed. After 24 h of stimulation, NO levels of LPS treated RAW264.7 cells were significantly higher. TE decreased NO production to 48.62 ± 2.2 and 35.47 ± 1.6% at 100 μg and 50 μg/ml concentration respectively (Fig 3). LPS induced NO was reduced to 62.24 ± 0.7% by dexamethasone at 100 μM concentration. In addition to ROS and RNS, NO is a signalling molecule implicated in widespread of pathological events like apoptosis gene expression, inflammation, enzyme regulation [48–49]. In response to inflammatory stimuli, high levels of NO are secreted that impart pro-inflammatory effects [48] and could act as an oxidant [46]. Plant derived products have intrinsic properties to combat the harmful effects of excessive NO production and thus have substantial interest in its prevention in humans. In this study, TE inhibited NO production in LPS-induced RAW264.7 cells. Previous studies have shown that TE has strong NO-scavenging properties [50].

**Fig 4:**
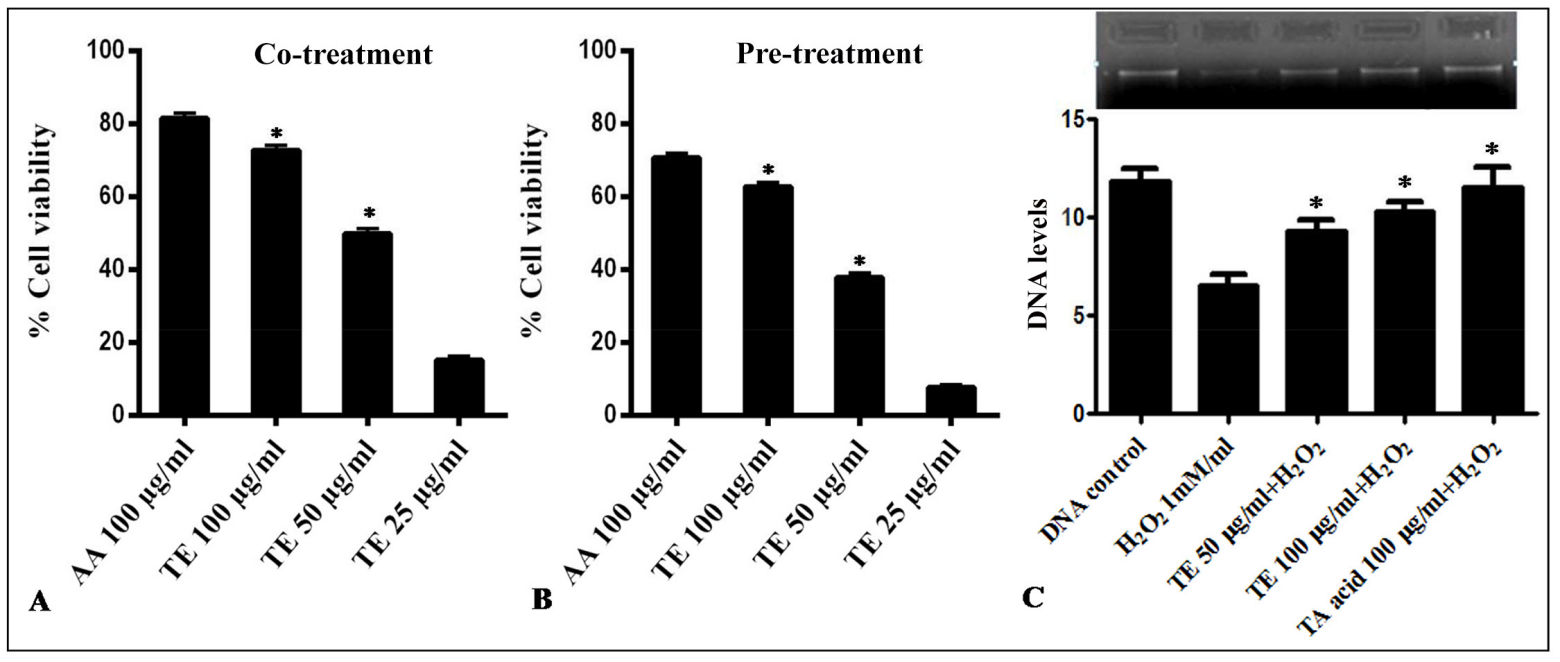
Radical scavenging effect of TE on HDF cells against H2O2-induced cell damage. (**A**) HDF cells were pre-treated with various concentrations of TE for 1 h and exposed to 10 μM H2O2, and further incubated for 24 h at 37°C. (**B**) HDFs were co-treated with various concentrations of TE and 10 μM H2O2, incubated for 24 h at 37°C. Cell viability was determined by MTT assay and the results expressed as mean ± SEM (n = 3). **(C) Protective effect of TE on DNA damage in HDF cells by release of OH radicals from Fenton reaction.** The DNA were isolated from HDF cells and treated with two concentrations of TE (50 and 100μg/mL), FeSO_4_ (2mM) and H_2_O_2_ (1 mM), incubated for 1 h at 37°C. DNA bands were resolved in 1% agarose gel stained with ethidium bromide. Densitometry analysis showing the protective effect of TE on H_2_O_2_-induced DNA damage. Values shown depict arbitrary units. Data is expressed as mean ± SEM (n = 3).

H_2_O_2_ is an oxidant that induces the generation of free radicals. In order to check the concentration of H_2_O_2_ required inducing reversible damage on fibroblasts, a modification of the protocol reported by Murrell *et al* [25] was implemented. MTT assay was employed to study the protection rendered by TE at different concentrations. Ascorbic acid and TE extract at the concentration of 100 μg/mL showed a significant protection of fibroblasts against hydrogen peroxide-induced cell membrane damage. The fibroblasts co-treated with the extract showed more protection than that of pre-treatment of TE and hydrogen peroxide (Fig 4A and 4B). However, TE could not regain the viability of the fibroblasts post-treatment (data not shown). Oxidation is generally considered to play an important role in various disorders in cells [51]. In this study, TE exhibited antioxidant activity and protected HDF cells from H_2_O_2_ damage. The results of the MTT assay indicated that TE could rescue HDF cells from oxidative injury induced by H_2_O_2_. The fibroblasts pre-treated and co-treated were protected from the damage suggesting that TE might protect the cell membrane of HDFs, thus limiting the damage induced by H_2_O_2_.

The skin of humans is amenable to different DNA-damaging factors of the environment and hence, needs several in-house functions to reduce, repair, and protect from such deleterious effects. Skin pigmentation, antioxidant enzymes, epidermal thickness, DNA repair and apoptosis are the mechanisms involved in offering skin protection. [52]. In the present study, TE showed strong scavenging effects on hydroxyl radicals and was further examined for its protective role against DNA damage due to generation of hydroxyl ions via Fenton reaction. The DNA treated with 2 mM FeSO_4_ and 1mM H_2_O_2_ lead to oxidation of DNA generating free hydroxyl ions from the fenton’s reaction. However, TE treatment showed a dose-related inhibition of DNA oxidation against OH radical induced damage at 100 and 50 μg/mL concentrations respectively (Fig 4C). Tannic acid also protected HDF from DNA damage (Fig 4C). Thus, TE ameliorated hydroxyl radical-stimulated DNA damage. The precursors and catalysts for Fenton’s reaction co-exist in the mitochondrial matrix that makes mitochondria an important site for sustained hydroxyl radical production during oxidative stress [53]. Exposure of DNA to Fenton reaction leads to DNA strand breaks and base modifications [54–55]. In the experimental study, the rate of DNA damage was typically reduced by TE, showing its protective effects against H_2_O_2_ damage.

**Fig 5:**
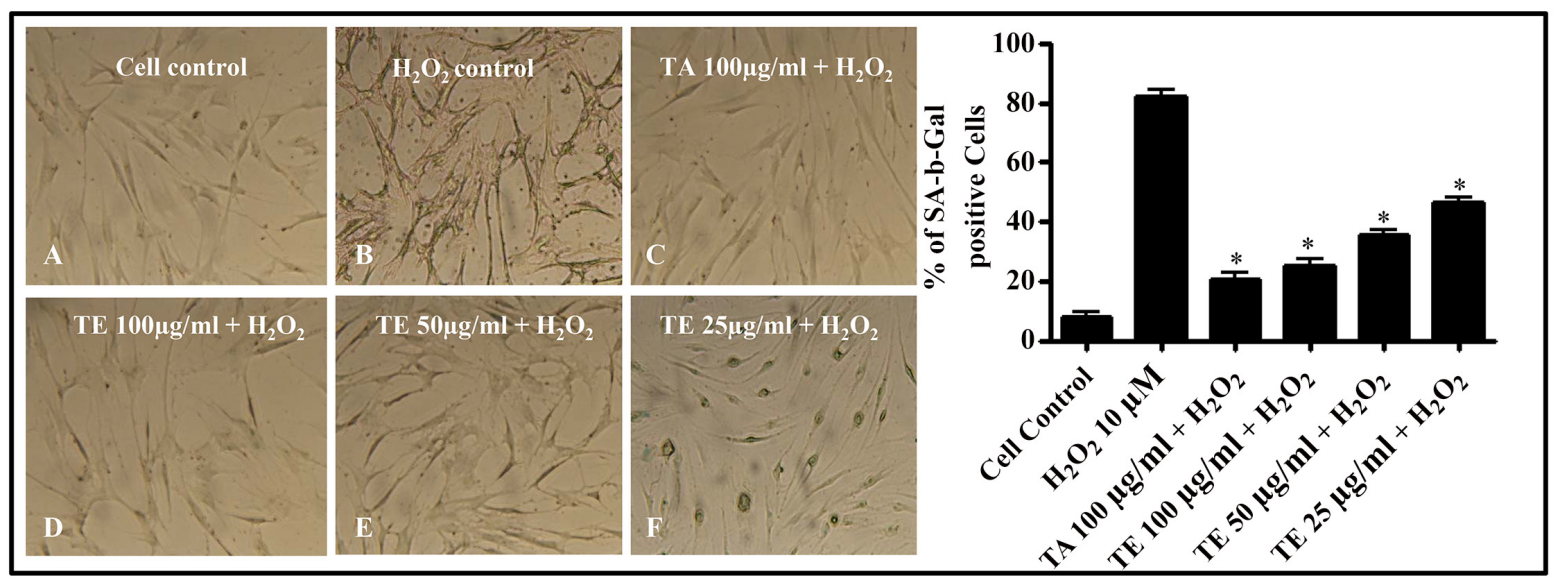
Effect of TE on cellular senescence in HDF cells. The cells were treated with two concentrations (50 and 100 μg/ml) of TE and H_2_O_2_ (10 μM), incubated for 1 h at 37°C. The cells were stained and incubated for 24 h at 37°C. The cells were counted under inverted microscope (10 ×). **(A)** Cell control **(B)** H_2_O_2-_treated cells **(C)** Cells treated with tannic acid (TA) (100 μg/ml) and H_2_O_2_ **(D)** Cells treated with TE (100 μg/ml) and H_2_O_2_ **(E)** Cells treated with TE (50 μg/ml) and H_2_O_2_. **(F)** Cells treated with TE (25 μg/ml) and H_2_O_2_. **(F)** Percentage reduction in SA-β-gal cells, expressed as mean ± SEM (n = 3) (*p* ≤ 0.01, p ≤ 0.05).

Cellular senescence plays a vital role in regulating cellular aging both *in vitro* and *in vivo* [56]. The aging cells are identified with increased lipofuscin granules and a distinct phenotype, which includes flat morphology and bulged cell size [56–57]. Significant changes are also observed at protein and gene levels [57]. Accumulation of β-galactosidase occurred in H_2_O_2_ induced HDF cells in the fibroblasts, appeared as bluish green cells under the microscope (Fig 5A–5G). TE exhibited protective effect on H_2_O_2_ induced HDF cells with a significant decrease in β-galactosidase stained cells. The cells treated with tannic acid, used as standard also protected the cells from H_2_O_2_ induced cellular senescence (Fig 5C). Cellular senescence is an indication of age-associated etiology [58]. H_2_O_2_ induces senescence-associated beta-galactosidase activity in HDF cells [59–60]. Thus TE protected the cells from H_2_O_2_-induced cellular senescence that proved its anti skin aging property.

**Fig 6:**
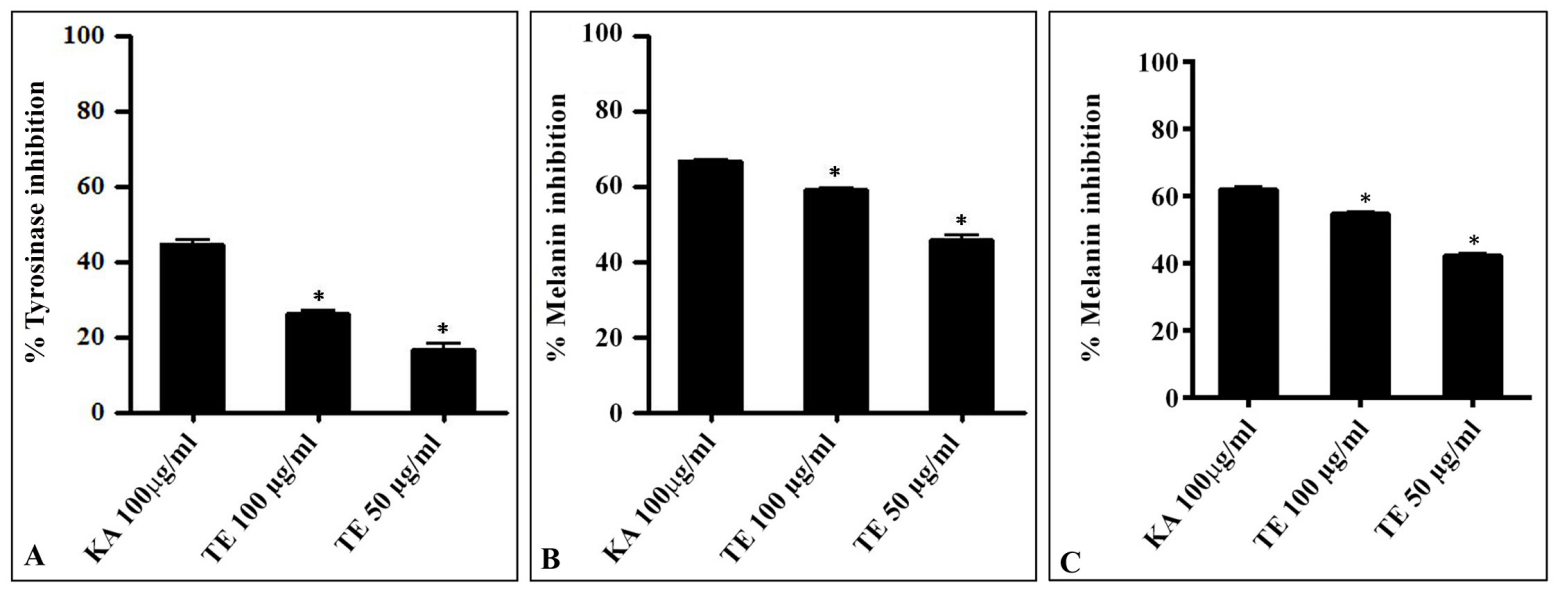
Anti-tyrosinase and melanin inhibitory properties of TE. The B16F10 cells were cultured in the 40mm petri plates (1×10^5^ cells/well) using the DMEM medium supplemented with 10% FBS for 24 h. **(A) Tyrosinase inhibition assay:** The cells were treated with different concentrations of TE and then incubated for 24 h with or without 100 nM α-MSH. The cells were lysed, centrifuged, and supernatant collected was monitored at 492 nm. Percentage of tyrosinase inhibition was calculated and expressed as mean ± SEM (n = 3) (*p* ≤ 0.01, p ≤ 0.05). **(B) Melanin inhibition/spitting assay:** The cells were treated with different concentrations of TE and 100 μM concentration of forskolin for the stimulation of melanin for 72 h and detached by 0.05% trypsin-EDTA. The cell pellets obtained were dissolved in 1 N NaOH for 1 h at 65°C and melanin content was measured at 405 nm. For % tyrosine inhibition and melanin content analysis, kojic acid (100 μM) was used as a positive control. Mean ± SEM (n = 3) (*p* ≤ 0.01, p ≤ 0.05). **(C) Melanin inhibition assay in Human melanoma (A-375) cells.** The experimental conditions remain same as above.

Melanogenesis involves the enzyme tyrosinase for catalyzing the oxidative reaction of tyrosine to dihydroxy-phenylalanine (DOPA) and Dopaquinone, a common intermediate to both eumelanogenic and pheomelanogenic pathways [61–63]. Conventionally, L-tyrosine and L-DOPA are regarded as positive regulators of melanogenesis [64]. Melanin possesses photoprotective properties; the cumulation of abnormal amounts of melanin in various skin parts leads to darkening or skin pigmentation that hampers the skin beauty. To evaluate the skin-whitening properties of TE, melanin spitting/inhibition, and anti-tyrosinase assays were performed. The mouse melanoma cells (B16F10) is widely used for investigating anti-tyrosinase and melanin inhibition properties of the plant extracts [65–67]. TE showed tyrosinase inhibition 26.17 ± 1.0% and 16.16 ± 2.3% at 100 μg/ml and 50 μg/mL respectively in B16F10 cells. Kojic acid (KA) was used as a positive control, showed 44.63 ± 1.2% at 100 μM concentration (Fig 6A). Earlier, ellagic acid inhibited UV irradiation induced skin pigmentation [65–67]. Previous literature on the inhibition of tyrosinase activity by several compounds led to the use of various inhibitors in cosmetics for the treatment of hyperpigmentation [68–69]. Similarly, in melanin inhibition assay, TE displayed significant melanin inhibition at 100 μg/ml (59.08 ± 0.7%) and 50 μg/mL (45.55 ± 2.0%) respectively in B16F10 cells. Kojic acid (KA) was used as a reference standard and it showed 66.71 ± 0.6% at 100 μM concentration (Fig 6B). In addition, the melanin inhibition assay was also performed in A-375 (Human melanoma) cells. TE showed dose-dependent melanin inhibition (Fig 6C). Several plant extracts like *Morus alba* L. (Moraceae) or *Glycyrrhiza glabra* (Leguminosae) have found their applications as skin lightening agents in cosmeticuticals [70–72].

**Fig 7:**
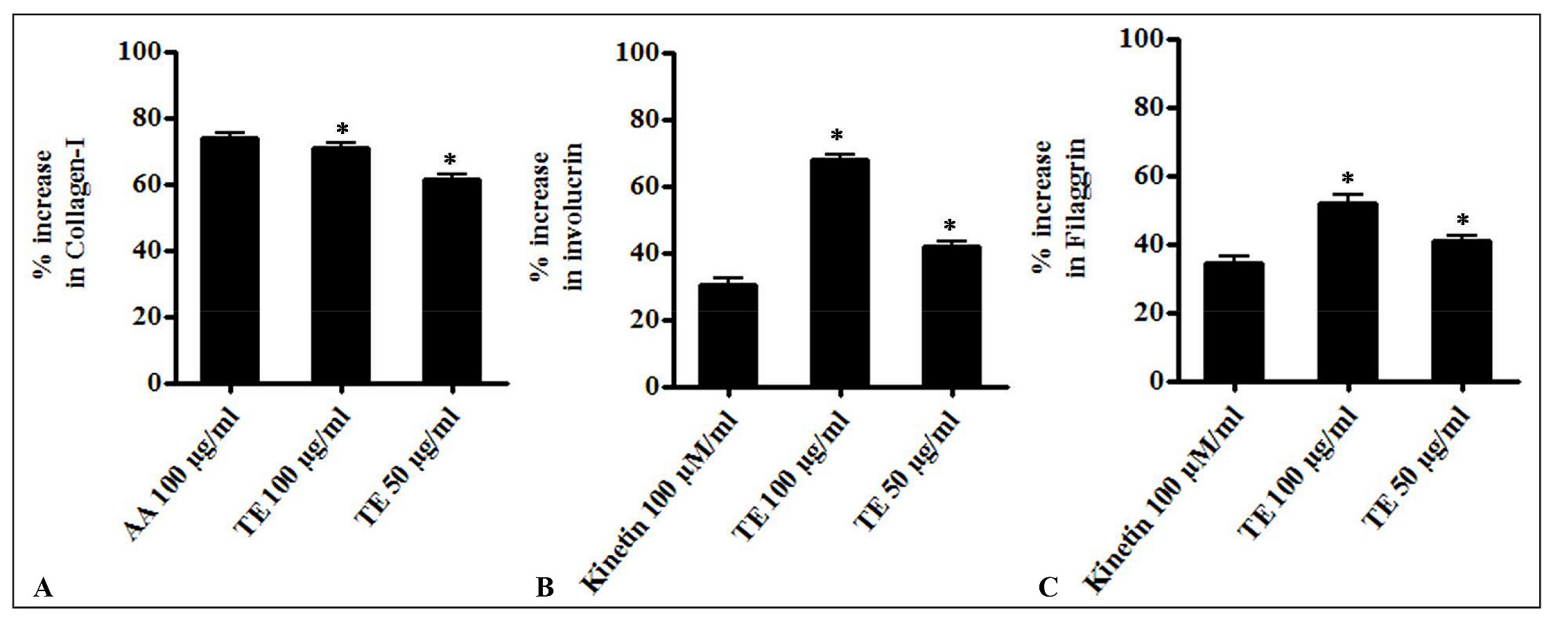
(A) Effect of TE on collagen-I production in HDF cells. The HDF cells were treated with two concentrations of TE (50 and 100μg/ml) and incubated for 96 h at 37°C. Collagen-I in cell supernatants were estimated by ELISA and results expressed as percentage of control. (B) Effect of TE on involucrin production in HaCaT cells. HaCaT were treated with two concentrations of TE (50 and 100 μg/ml) and incubated for 96 h at 37°C. Involucrin content in cell supernatants was estimated by ELISA and results expressed as percentage of control. (C) Effect of TE on filaggrin production in HaCaT cells. HaCaT were treated with two concentrations of TE (50 and 100 μg/ml) and incubated for 96 h at 37°C. Filaggrin content in cell supernatants was estimated by ELISA and results expressed as percentage of control. Mean ± SEM (n = 2); (*p* ≤ 0.01, p ≤ 0.05).

Human dermal fibroblasts continuously produce products related to extracellular matrix and collagen-I, is one such classical product. To evaluate the effect of TE on collagen-I production in fibroblast cells, ELISA was performed. HDF treated with TE showed an increase in collagen-I production by 70.72 ± 2.3% and 58.24 ± 4.6% at 100 μg and 50 μg/mL respectively. Ascorbic acid increased collagen-I production by 73.67 ± 2.2% in HDF cells (Fig 7A). In addition, TE treated HDF cells producing collagen-I stained pink as detected by Sirus red staining (S6 Fig). Keratinocytes, apart from providing the structural integrity, also secrete out various substances like cytokines, hormones, and neurotransmitters [3]. Differentiating keratinocytes express cytokeratins and accessory proteins such as involucrin, filaggrin, loricrin, cystatin, envoplakin, periplakin etc. in progressing layers to build the epidermal barrier [3]. ELISA measurements revealed a steady increase in involucrin content in TE treated HaCaT cells after 4 days. TE increased involucrin content by 67.61 ± 2.1% and 41.95 ± 1.9% at 100 and 50 μg/mL respectively (Fig 7B). Kinetin increased the involucrin content by 30.30 ± 2.8% at 100 μM concentration. TE increased filaggrin by 51.91 ± 3.5% and 41.01 ± 2.1% at 100 and 50 μg/mL respectively (Fig 7C). Kinetin increased the filaggrin content by 34.38 ± 3.0% at 100 μM concentration.

**Fig 8:**
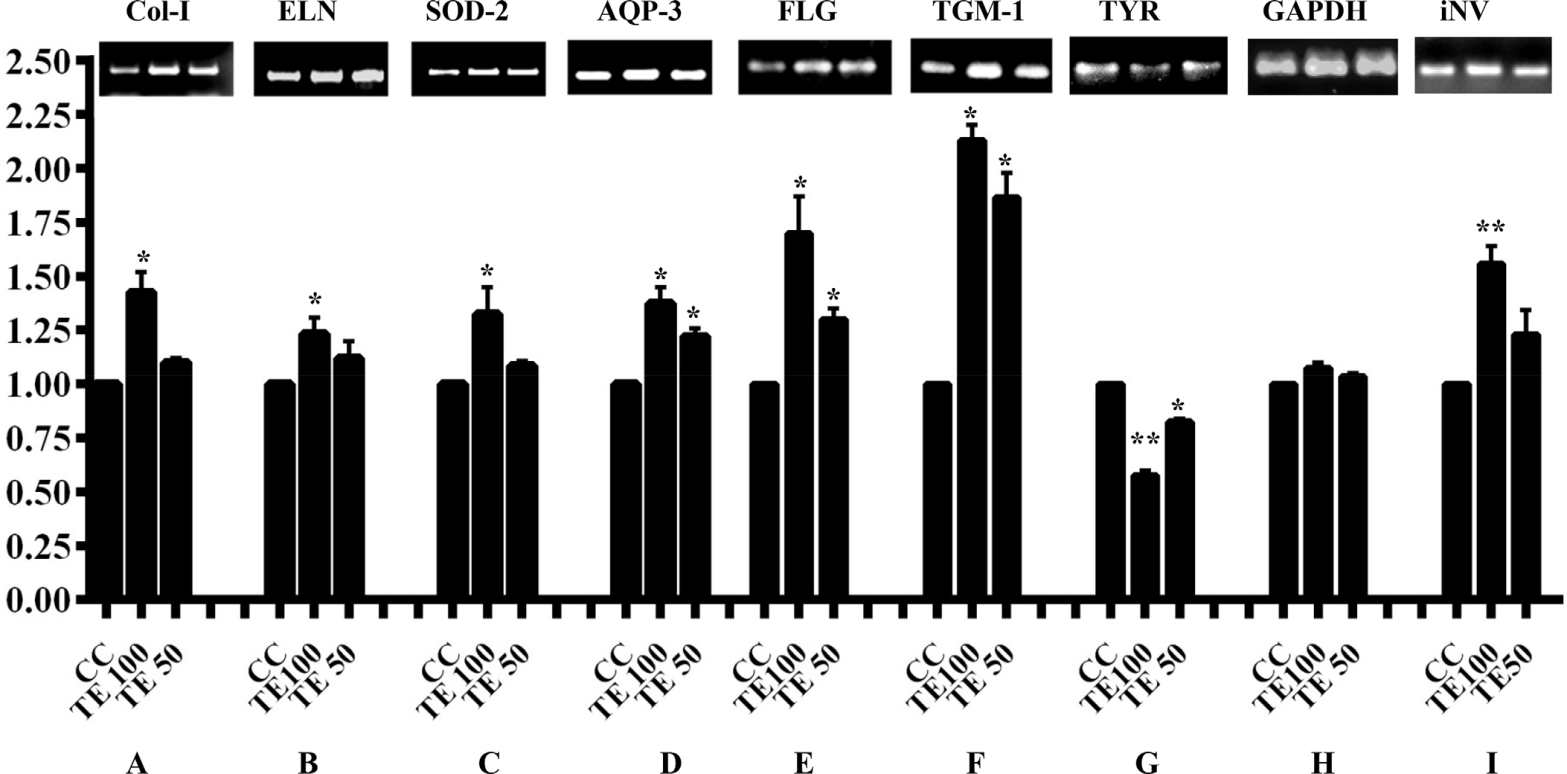
Effect of TE on expression of selected genes involved in biological functions related to aging. HDF and/or HaCaT cells were treated with different doses of TE for 24 h at 37°C. RNA was then isolated and qPCR carried out using oligo-dT primers for first strand synthesis followed by gene specific primers as described in the section 2.14. The gene expression was quantified and normalized to GAPDH and the data represented as mean ± SD (n = 2). The image shown is a representative from among two replicates. * indicate P < 0.05 and ** indicate P < 0.01. (**A**) Collagen-I gene expression (**B**) Elastin gene expression (**C**) SOD-2 gene expression (**D**) AQP-3 gene expression **(E)** Filaggrin gene expression **(F)** Transglutaminase (TGM1) gene expression **(G)** Tyrosinase gene expression **(H)** GAPDH gene expression (**I**) Involucrin gene expression.

In dermis, the reduction of skin structural proteins can lead to several signs of aging [73–74]. To further establish the skin protective role of TE, the mRNA levels for skin-related proteins/markers were checked. TE at 100 and 50 μg/ml increased the gene expression of collagen-I, elastin, SOD-2, AQP-3, transglutaminase, involucrin, filaggrin in HDF or HaCaT cells. However, the tyrosinase gene expression decreased upon treatment with TE at 100 and 50 μg/ml in B16F10 cells. The Ct values were normalized to that of GAPDH and given in the (Fig 8A–8I). The amplified products stained with ethidium bromide confirmed the results of qPCR (Fig 8). Collagen, one of the structural proteins of the skin, is the major constituent of connective tissue, hair, and nails [75]. It helps in maintaining the elasticity, firmness and flexibility of the skin. Elastin, a predominant protein in the connective tissue provides elasticity to the skin and lungs [75, 76]. Involucrin one of the important markers of the skin helps in protecting the skin corneocytes by formation of the cell envelope [77]. TE-treated HDF or HaCaT cells increased the synthesis of collagen-I and involucrin respectively. The expression profile of collagen and elastin were up-regulated in TE treated HDF cells. The results highlight the role of TE in increasing collagen and elastin gene expression. AQP-3 in the skin of human beings is age-related and decreases in level occur with aging [78]. In dermal pathophysiological course, AQP-3 can transport water and glycerol to epidermis, so AQP-3 is significant to epidermal barrier and water retention. AQP-3 gene expression was up-regulated by TE in HaCaT cells, indicating that TE can function as a skin protectant by hydrating the skin cells. The molecules such as thiols, DNA, and mitochondrial proteins are regarded most responsive to modification by oxidative stress [79]. Stress on epidermal skin leads to generation of several metabolites that scavenge free radicals and stimulate antioxidative enzymes, including superoxide dismutase [80]. SOD-2 is a cellular antioxidant present in mitochondrial matrix and offers defence against superoxide anions, which are the by-products of oxidative phosphorylation [81]. The present study showed that TE stimulates SOD-2 expression in HDF cells, thus enabling them to combat oxidative stress.

Filaggrin, a protein that binds to keratin fibers in epithelial cells plays a vital role in epidermal homeostasis, release of free amino acids, and helps in water retention [82]. The filaggrin gene expression was up-regulated upon treatment with different concentrations of TE in HaCaT cells, indicating the role of TE in skin moisturization. Transglutaminase, a calcium-dependent enzyme catalyzes an intermolecular peptide bond formation between proteins and has an essential role in formation of cornified cell envelope [83]. The transglutaminase gene (TGM1) expression was up-regulated in HaCaT cells after treatment with TE at two different concentrations indicating pore reduction/or skin tightening. Tyrosinase catalyzes tyrosine to DOPA and Dopaquinone thus playing a vital role in melanogenesis, which, is explored by researchers for screening various melanin inhibitors [68–72]. The tyrosinase gene expression was down-regulated by TE in B16F10 cells, suggesting its role in skin-whitening.

Triphala (TE) has become a potential candidate for skin care and protection through its antioxidant and anti-inflammatory properties. However, no studies have demonstrated the skin protecting benefits of TE using *in vitro* skin models. The results highlight the benefits of Triphala (TE) associated with its potential antioxidant property, radical-scavenging ability, delaying cellular senescence, building structural proteins such as collagen and elastin, restoring skin barrier function along with providing hydration to the skin.

## Conclusion

This article for the first time reports the protective effects of Triphala (TE) on Human skin cells *in vitro*. Triphala has potential antioxidant property and it acts as a skin-protective ingredient by re-building skin structural proteins and stimulating selective youth genes. The results obtained however, needs further detailed clinical investigation.

## Supporting Information

S1 Fig LC-MS spectra of *Terminalia chebula* extract. The arrow indicates the identified compounds.
(TIF)

S2 Fig LC-MS spectra of *Emblica officinalis* extract. (TIF)

S3 Fig LC-MS spectra of *Terminalia bellerica* extract. (TIF)

S4 Fig Docking studies of major constituents of Triphala (Gallic acid, Ellagic acid, and Chebulinic acid) with involucrin, filaggrin, & collagen-I. a-c) Involucrin d-f) Filaggrin g-i) Collagen-I. The black arrow indicates the docked compound.
(TIF)

S5 Fig Effect of TE on HaCaT and HDF cells a) Cytotoxicity of Triphala (TE) on HaCaT and HDF cells b) Untreated HaCaT cells (Control) c) TE (50 μg/ml) treated HaCaT cells d) TE (100 μg/ml) treated HaCaT cells e) HDF cells without treatment (Control) f) TE (50 μg/ml) treated HDF cells g) TE (100 μg/ml) treated HDF cells. (TIF)

S6 Fig Collagen-I staining in HDF cells a) Control b) Ascorbic acid c) TE (50 μg/ml) treated HDF cells d) TE (100 μg/ml) treated HDF cells. The pink color indicates the collagen-I production in HDF cells.
(TIF)

S1 Table Protein-Ligand binding affinity in Kcal/mol. (DOC)

S1 Text Raw/Experimental data for protective effects of TE on HDF and/or HaCaT cells a) Cytotoxicity of TE on HaCaT and HDF cells b) RBC hemolysis assay c) Reducing power assay d) Radical scavenging assay e) Collagen-I ELISA assay. (XLSX)

## Abbreviations

AQP: Aquaporin
CE: Collision energy
DMEM: Dulbecco’s modified Eagle’s medium
DP: Declustering potential
HaCaT: Human keratinocytes
HDF: Human dermal fibroblast
HPLC: High performance liquid chromatography
LC: Liquid chromatography
LPS: Lipopolysaccharide
MEM: Minimum essential medium
MS: mass spectrophotometer
NO: Nitric oxide
RNS: Reactive nitrogen species
ROS: Reactive oxygen species
SOD: Superoxide dismutase
TE: Triphala extract
