# Supplementary figures and images for: Protective Effects of Triphala on Dermal Fibroblasts and Human Keratinocytes
Source: PLoS One. 2016 Jan 5;11(1):e0145921. doi: 10.1371/journal.pone.0145921 (PMC4711708; doi:10.1371/journal.pone.0145921)

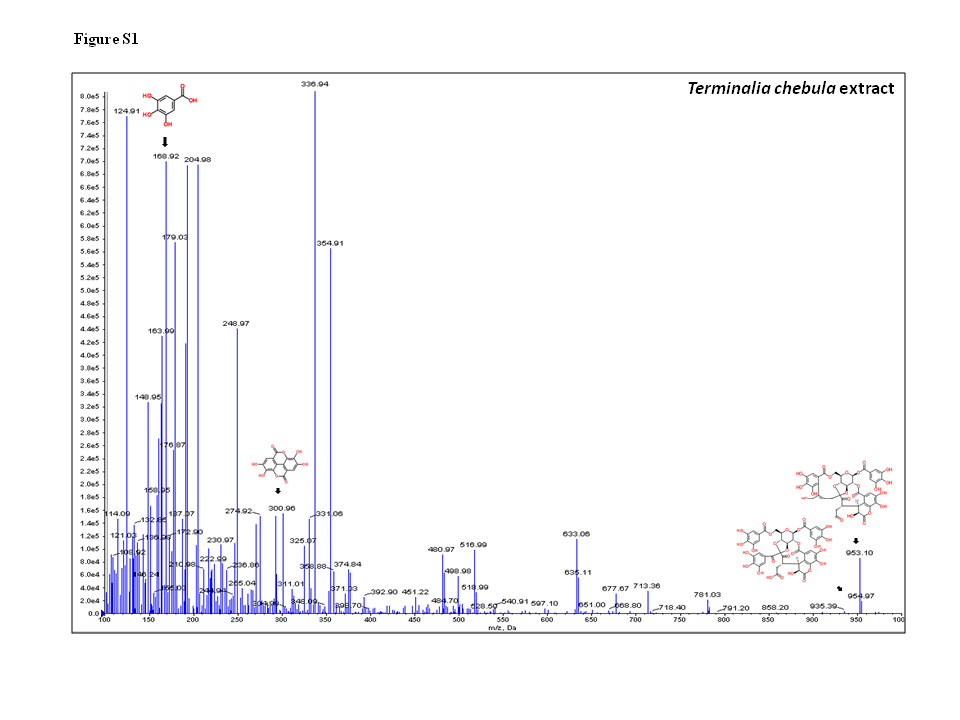

Supplement: S1 Fig — The arrow indicates the identified compounds. (TIF) [file pone.0145921.s001.TIF]

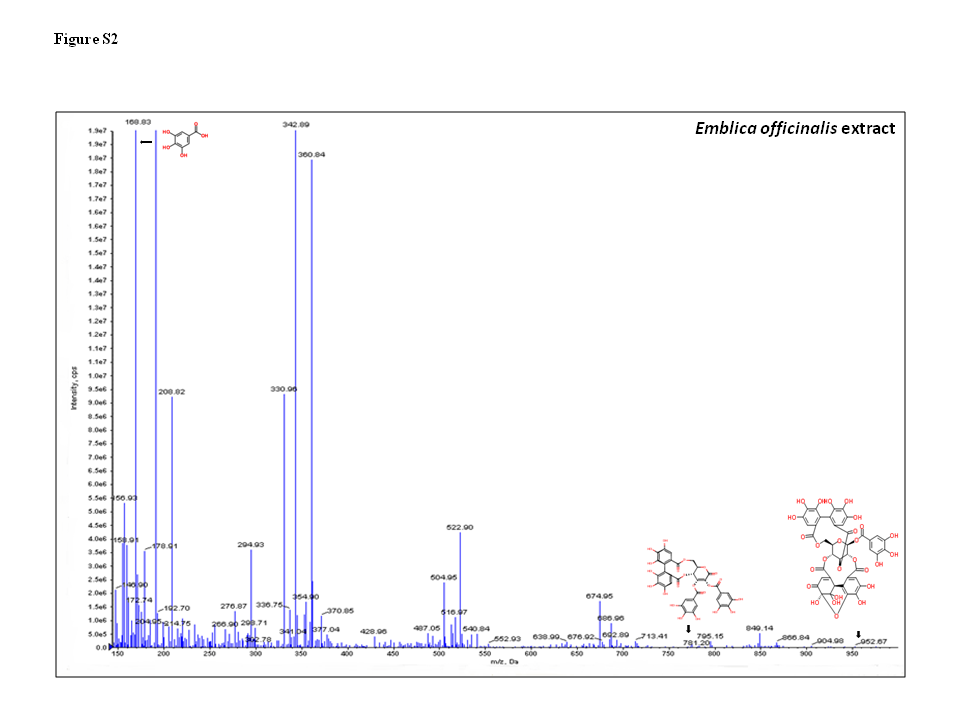

Supplement: S2 Fig — (TIF) [file pone.0145921.s002.TIF]

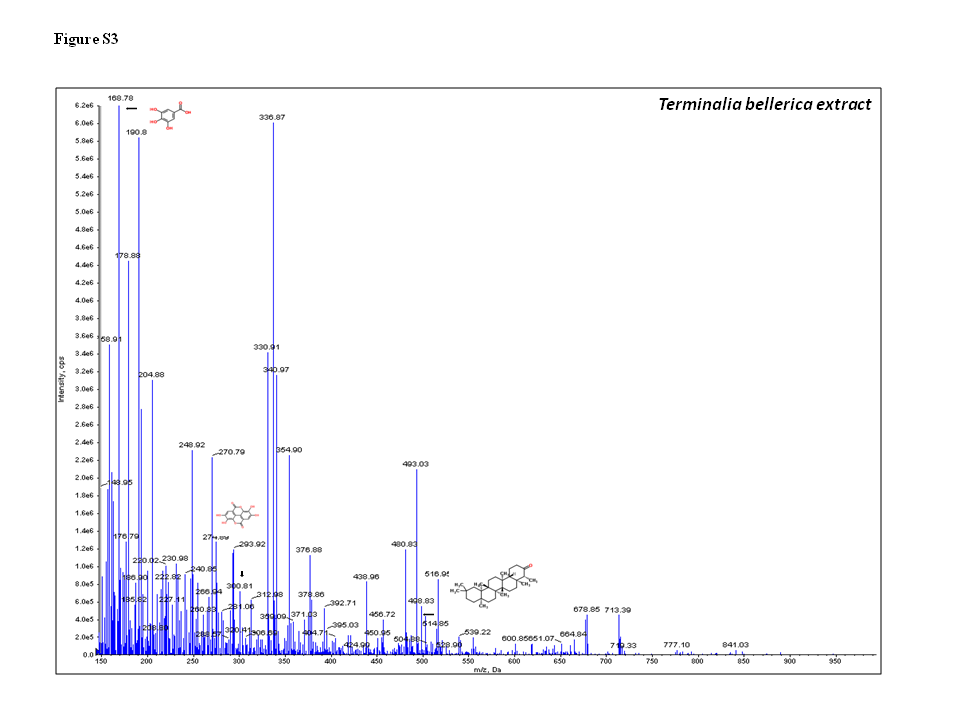

Supplement: S3 Fig — (TIF) [file pone.0145921.s003.TIF]

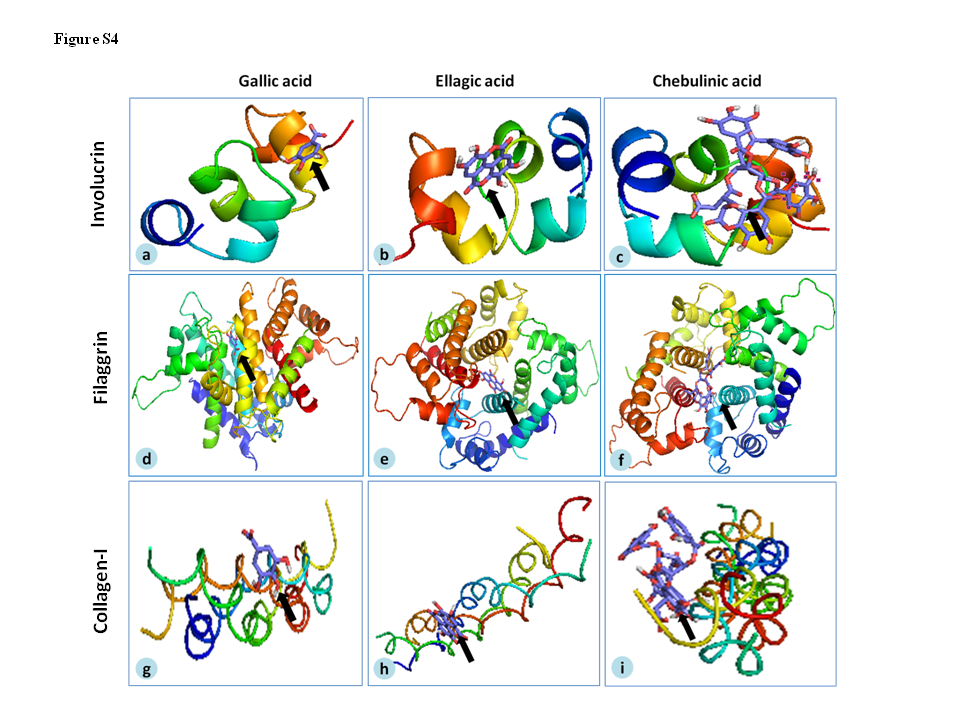

Supplement: S4 Fig — a-c) Involucrin d-f) Filaggrin g-i) Collagen-I. The black arrow indicates the docked compound. (TIF) [file pone.0145921.s004.TIF]

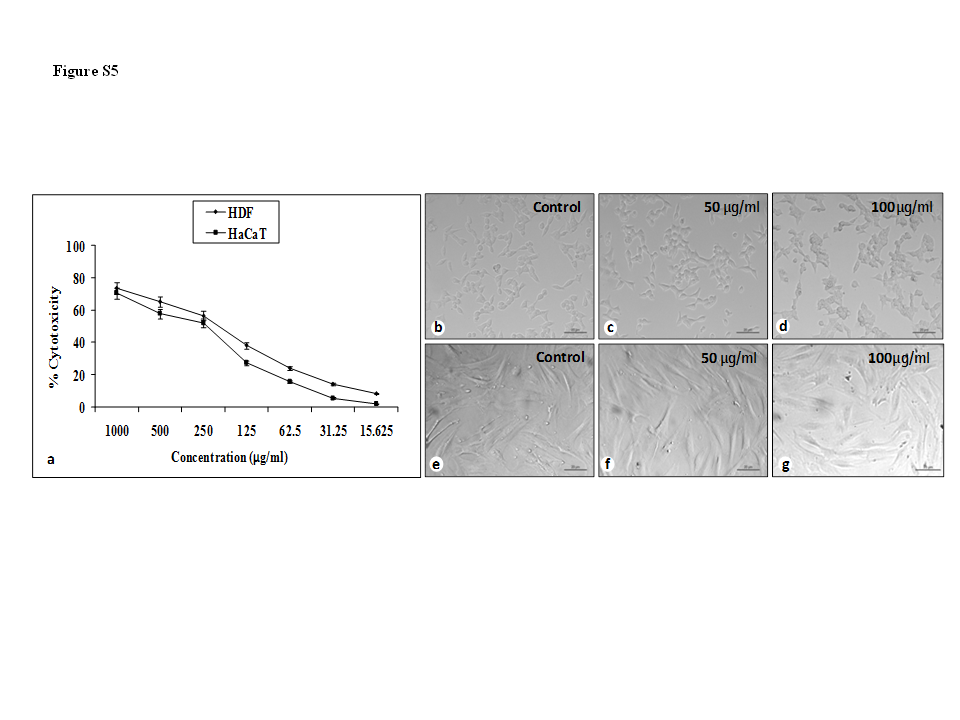

Supplement: S5 Fig — (TIF) [file pone.0145921.s005.TIF]

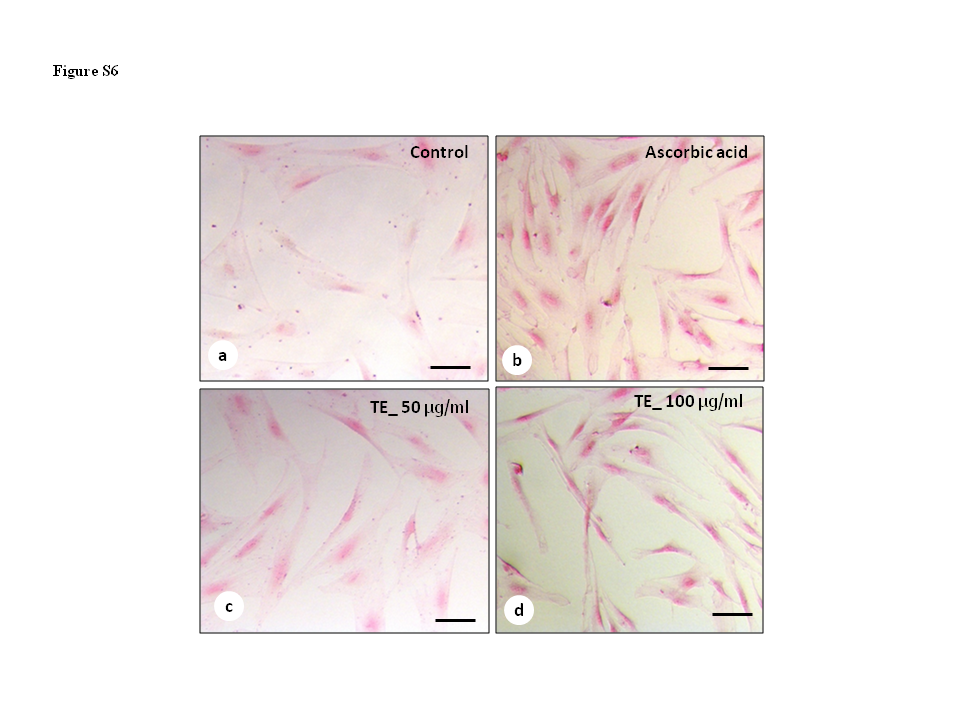

Supplement: S6 Fig — The pink color indicates the collagen-I production in HDF cells. (TIF) [file pone.0145921.s006.TIF]
