# Supplementary material for: Protective Effects of Triphala on Dermal Fibroblasts and Human Keratinocytes
Source: PLoS One. 2016 Jan 5;11(1):e0145921. doi: 10.1371/journal.pone.0145921 (PMC4711708; doi:10.1371/journal.pone.0145921)
Supplement: S1 Table — (DOC) [file pone.0145921.s007.doc]

**Supplementary Data**

**S1 Table Protein**-**Ligand binding affinity in Kcal/mol**

| **S.No.** | **Protein** | **Ligand** | **Binding affinity**  **(Kcal/mol)** |
| --- | --- | --- | --- |
| 1. | AQP-3 | Gallic acid | -5.9 |
|  |  | Ellagic acid | -8.7 |
|  |  | Chebulinic acid | -9.3 |
| 2. | SOD-2 | Gallic acid | -5.0 |
|  |  | Ellagic acid | -6.1 |
|  |  | Chebulinic acid | -7.9 |
| 3. | Collagen | Gallic acid | -3.8 |
|  |  | Ellagic acid | -5.2 |
|  |  | Chebulinic acid | -6.4 |
| 4. | Transglutaminase | Gallic acid | -6.1 |
|  |  | Ellagic acid | -8.9 |
|  |  | Chebulinic acid | -9.6 |
| 5. | Tyrosinase | Gallic acid | -6.1 |
|  |  | Ellagic acid | -7.7 |
|  |  | Chebulinic acid | -9.0 |
| 6. | Filaggrin | Gallic acid | -6.0 |
|  |  | Ellagic acid | -8.2 |
|  |  | Chebulinic acid | -11.0 |
| 7. | Involucrin | Gallic acid | -4.7 |
|  |  | Ellagic acid | -6.0 |
|  |  | Chebulinic acid | -6.5 |
